# Silent Sentence Completion Shows Superiority Localizing Wernicke’s Area and Activation Patterns of Distinct Language Paradigms Correlate with Genomics: Prospective Study

**DOI:** 10.1038/s41598-017-11192-2

**Published:** 2017-09-21

**Authors:** Kamel El Salek, Islam S. Hassan, Aikaterini Kotrotsou, Srishti Abrol, Scott H. Faro, Feroze B. Mohamed, Pascal O. Zinn, Wei Wei, Nan Li, Ashok J. Kumar, Jeffrey S. Weinberg, Jeffrey S. Wefel, Shelli R. Kesler, Ho-Ling Anthony Liu, Ping Hou, R. Jason Stafford, Sujit Prabhu, Raymond Sawaya, Rivka R. Colen

**Affiliations:** 1 Section of Neuroradiology, Department of Diagnostic Radiology, The University of Texas MD Anderson Cancer Center, Houston, Texas United States; 2 Department of Radiology, Temple University, Philadelphia, Pennsylvania United States; 3 Department of Radiology, Thomas Jefferson University Hospital, Philadelphia, Pennsylvania United States; 4 Department of Neurosurgery, Baylor College of Medicine, Houston, Texas United States; 5 Division of Quantitative Sciences, Department of Biostatistics, The University of Texas MD Anderson Cancer Center, Houston, Texas United States; 6 Department of Neurosurgery, The University of Texas MD Anderson Cancer Center, Houston, Texas United States; 7 Section of Neuropsychology, Department of Neuro-Oncology, Division of Cancer Medicine, The University of Texas MD Anderson Cancer Center, Houston, Texas United States; 8 Department of Imaging Physics, The University of Texas MD Anderson Cancer Center, Houston, Texas United States; 9 Department of Cancer Systems Imaging, The University of Texas MD Anderson Cancer Center, Houston, Texas United States

## Abstract

Preoperative mapping of language areas using fMRI greatly depends on the paradigms used, as different tasks harness distinct capabilities to activate speech processing areas. In this study, we compared the ability of 3 covert speech paradigms: Silent Sentence Completion (SSC), category naming (CAT) and verbal fluency (FAS), in localizing the Wernicke’s area and studied the association between genomic markers and functional activation. Fifteen right-handed healthy volunteers and 35 mixed-handed patients were included. We focused on the anatomical areas of posterosuperior, middle temporal and angular gyri corresponding to Wernicke’s area. Activity was deemed significant in a region of interest if P < 0.05. Association between fMRI activation and genomic mutation status was obtained. Results demonstrated SSC’s superiority at localizing Wernicke’s area. SSC demonstrated functional activity in 100% of cancer patients and healthy volunteers; which was significantly higher than those for FAS and CAT. Patients with 1p/19q non-co-deleted had higher extent of activation on SSC (P < 0.02). Those with IDH-1 wild-type were more likely to show no activity on CAT (P < 0.05). SSC is a robust paradigm for localizing Wernicke’s area, making it an important clinical tool for function-preserving surgeries. We also found a correlation between tumor genomics and functional activation, which deserves more comprehensive study.

## Introduction

Pre-surgical brain mapping has become part of the standard of care for patients with brain tumors^1^. One of the most important tools for pre-surgical brain mapping is functional MRI (fMRI), which relies on blood oxygen level-dependent (BOLD) signal to obtain rapid and repeated images of the brain while the patient performs special tasks^2^. To map cortical speech areas using fMRI, a well-tailored task is performed to ensure the activation of a specific eloquent region. A plethora of speech paradigms have been developed, with reliable results, for mapping Broca’s area. However, these tasks have shown inconsistent results when mapping Wernicke’s area^3,4^. Localization of Wernicke’s area is challenging due to the relative variation in its anatomical location^5^ and because the complexity of speech processes extend beyond typical anatomical locations of Broca’s and Wernicke’s areas^6^. Further, receptive speech processing has been postulated to be a function of intertwined neuronal mechanisms that involve decision making, attention span, and working memory^4,7^. This explains why the commonly used paradigms have shown variability and a lack of consistency for pre-surgical mapping purposes^4,8,9^.

To date, two studies, Zaca *et al*. and Ashtari *et al*.^3,4^, have evaluated silent sentence completion (SSC) in localizing areas of receptive speech processes. However, neither of these studies had a sufficiently large population to generalize their results nor did they included brain tumor patients, whose performance can be impaired. The importance of including brain cancer patients in any study evaluating fMRI paradigms is the fact that tumors such as gliomas have been associated with various degrees of neurovascular uncoupling (NVU), which decreases the BOLD contrast needed to detect active areas^10^. Thus, evaluating language tasks in such patients is important for clinical implementation. Further, the aforementioned studies only included right-handed participants.

Recent studies have shown that there is a strong correlation between certain genetic markers and perfusion, specifically relative cerebral blood volume (rCBV)^11,12^. Furthermore, certain key genomic markers have been associated with changes in tumoral and peritumoral microenvironement^11^, which might impact the BOLD signal and thus final fMRI result. Given that some of these markers carry important prognostic significance^13^, variations in patterns of fMRI activation seen in cancer patients should be evaluated in the context of differential genomic aberations.

Therefore, at its core, our study aims to determine the adequacy and robustness of SSC in localizing Wernicke’s area compared to the most clinically used language tasks, i.e. CAT (category naming) and FAS (Word generation)^4^. Furthermore, we attempt to determine the association between functional activation patterns and signal intensity with the following key genetic markers: (i) tumor suppressor genes commonly mutated in gliomas (tumor protein 53 (*TP53*), phosphatase and tensin homolog (*PTEN*)), (ii) epidermal growth factor receptor (*EGFR*) which is associated with angiogenesis, (iii) genetic alterations that characterize low grade gliomas and determine their subtype (isocitrate dehydrogenase 1 and 2 (*IDH-1*/2), *1p/19q* co-deletion), (iv) downstream substrates and epigenetric events associated with treatment response in gliomas (*S-100*, and O^6^-methylguanine-DNA methyltransferase (*MGMT*) promoter methylation).

## Methods

### Subjects

#### Healthy Participants

We obtained approval from the institutional review board of The University of Texas MD Anderson Cancer Center (UTMDACC) (Houston, Texas) to perform this HIPAA-compliant prospective study (2011-0543-09), and all participants provided written informed consent prior to inclusion into the study. All methods were performed in accordance with the approved guidelines. We recruited 15 healthy participants between February 2014 and June 2015, all of whom were right-handed (7 males and 8 females; mean age, 38.8 years; median age, 37; range, 23–66 years) (Table 1). Healthy participants were recruited from a database of hospital volunteers. Subjects were initially contacted via email, a telephone screening was then performed to ensure the following inclusion criteria: ≥18 years, native English speaker, no prior brain surgeries, no neuropsychological disorders, and no contraindications to MRI. All participants underwent SSC; however, two patients did not undergo CAT and one did not undergo FAS due to technical difficulties at the time of scanning.

**Table 1:** Demographic distribution of healthy volunteers and activity on SSC, FAS, and CAT.

Age, years	Sex	Handedness	SSC	FAS	CAT
57	F	R	Positive	Negative	Negative
45	M	R	Positive	Negative	Negative
50	F	R	Positive	Negative	Positive
28	F	R	Positive	Positive	Negative
23	M	R	Positive	Positive	Negative
25	M	R	Positive	Negative	Negative
27	M	R	Positive	Negative	Negative
29	M	R	Positive	Negative	Negative
31	M	R	Positive	Negative	Negative
33	M	R	Positive	Positive	Positive
50	F	R	Positive	Positive	NT
42	F	R	Positive	Negative	NT
39(a,b)	F	R			
a)R temporal			Positive	Negative	Negative
b)L temporal			Positive	Negative	Negative
37	F	R	Positive	Positive	Negative
66	F	R	Positive	NT	Positive

NT: Not tested.

#### Cancer Patients

Approval from UTMDACC institutional review board (PA15-0029) was obtained for this HIPAA-compliant prospective study, and all participants provided written informed consent prior to inclusion into the study. All methods were performed in accordance with the approved guidelines. All cancer patients undergoing preoperative fMRI for brain mapping at UTMDACC from 8/1/14 to 12/31/2015 had SSC added to their study. The cancer cohort included 35 subjects (22 males and 13 females; mean age, 42.5 years; median age, 43; range, 18–69 years). The cohort included 26 right-handed, 5 left-handed, and 4 ambidextrous individuals (Table 2). Patients’ pathological disease types and grades are listed in Table 3. The inclusion criteria were as follows: 18 to 75 years of age, no contraindications to MRI, and evidence of a brain lesion on prior imaging. Ability to perform tasks accurately was based on the neuropsychology team’s overall assessment of their cognitive profile and behavioral observations. Neuropsychlogical testing was performed in clinic and directly before scanning. Handedness was determined via the Edinburgh handedness inventory. Histopathological diagnoses and grades were obtained from the available postoperative pathological reports^14^.

**Table 2:** Patients’ clinical and demographic characteristics and findings on SSC, FAS, and CAT.

Age, years	Sex	Handedness	Tumor location	Histology/grade	SSC	FAS	CAT	Genetic markers
36	F	R	L frontal	GBM/4	Positive	Negative	Negative	p53, EGFR, PTEN IHC+
32	M	R	L parietal	Oligo-dendroglioma/3	Positive	Negative	Negative	*1p19q* co-deletion, EGFR IHC+, IDH1 exon 4 mutation
67	M	R	L frontal	GBM/4	Positive	Positive	Positive	EGFR IHC+
54	F	L	L parietal	GBM/4	Positive	Positive	Negative	EGFR IHC+
51	M	R	L insula	Anaplastic oligo-dendroglioma/3	Positive	Positive	Positive	*1p/19q* co-deletion, IDH1 (R132H) IHC+
53	M	R	L posterior temporal	GBM/4	Positive	Negative	Negative	p53 IHC+, EGFR IHC+
18	F	R	L frontal	Mixed oligo-astrocytoma/3	Positive	Negative	Negative	IDH1 (R132H) IHC+, EGFR, PTEN IHC+
69	F	R	L inferior temporal	GBM/4	Positive	Negative	Negative	EGFR IHC+, *MGMT* promoter methylation
50	M	M	L frontal + temporal	GBM/4	Positive	Negative	Negative	p53 IHC+
37	M	R	L temporal	Oligo-dendroglioma/3	Positive	Negative	Negative	*IDH1* exon 4 mutation, 1p/19q co-deletion
65	F	R	L parietal + angular gyri	GBM/4	Positive	Negative	Negative	EGFR IHC+, PTEN loss of wild-type expression
45	M	R	L insula + frontal operculum	GBM/4	Positive	Negative	Negative	None
19	M	R	L parietotemporal	GBM /4	Positive	Negative	Negative	*TP53* exon 8 mutation, *IDH1* exon 4 mutation
33	M	R	L frontal, + L insula, + L temporal	Oligo-dendroglioma/3	Positive	Negative	Negative	*1p/19q* co-deletion, IDH1 (R132H) IHC+
18	F	R	R frontal + L frontal	Oligo-dendroglioma/2	Positive	Negative	Negative	*1p/19q* co-deletion, IDH1 (R132H) IHC+
39	M	M	R insula, frontal, + temporal	Oligo-dendroglioma/2	Positive	Negative	Negative	*1p/19q* co-deletion, IDH1 (R132H) IHC+
62	M	M	R frontal + insula	GBM/4	Positive	Negative	Negative	p53 IHC+
52	M	R	L caudate nucleus, internal capsule, + L temporal	GBM/4	Positive	Negative	Negative	p53 IHC+
43	M	R	L frontal	Astrocytoma/2	Positive	Positive	Positive	IDH (R132H) IHC+, p53 IHC+
43	M	L	R parietal	GBM/4	Positive	Negative	Negative	p53, S-100 IHC+
51	F	R	L frontotemporal	GBM/4	Positive	Negative	Negative	*S100* IHC+
51	F	R	L frontal, insula, +anterior temporal	GBM/4	Positive	Negative	Negative	*TP53* exon 7 mutation
28	F	L	L frontal	Astrocytoma/3	Positive	Positive	Positive	IDH-1 R132H) IHC+, EGFR IHC+
50	M	R	R + L frontal	GBM/4	Positive	Positive	Positive	p53, PTEN, EGFR IHC+
31	F	R	Superior L temporal	Ganglioglioma/2	Positive	Negative	Negative	None
62	M	L	L temporal	GBM/4	Positive	Negative	Negative	PTEN, EGFR, P53 IHC+
54	M	R	R frontal + L temporal	GBM/4	Positive	Negative	Negative	p53 IHC+
35	M	L	R occipito-temporal + bifrontal	Astrocytoma/3	Positive	Negative	Negative	IDH-1 (R132H) IHC+
25	F	R	R frontal	Oligo-dendroglioma/2	Positive	Positive	Positive	*1p/19q* co-deletion,IDH-1 (R132H) IHC+
35	M	R	L posterior temporal	Oligo-dendroglioma	Positive	Positive	Negative	None
49	F	R	L temporal	Oligo-dendroglioma	Positive	Positive	Negative	*1p/19q*, IDH-2 p.515 G > A IHC+
18	M	R	L temporal	Astrocytoma	Positive	Negative	Negative	p53 IHC+
33	M	R	R superior, middle, +inferior frontal gyri	Astrocytoma	Positive	Positive	Positive	IDH1 (R132H) IHC+
48	M	R	L frontal	Diffuse glioma	Positive	Positive	Positive	IDH1 (R132H) IHC+, *1p/19q* co-deletion
32	F	M	L frontal	Astrocytoma	Positive	Negative	Negative	IDH1 (R132H) IHC+

R: right, L: left, IHC: Immunohistochemistry, GBM: glioblastoma.

**Table 3:** Pathological types and grades of tumors.

Pathological type	Grade			
	1	2	3	4
GBM	—	—	—	17
Oligo-dendroma	1	4	4	—
Astrocytoma	—	4	2	—
Mixed oligoastrocytoma	—	—	1	—
Glioma	—	2	—	—

GBM: glioblastoma.

### Imaging techniques

Functional and structural images were acquired using a General Electric 3.0 Tesla Discovery MR750 MR scanner (GE Healthcare, Waukesha, WI, USA) with a 32-channel birdcage head coil (healthy volunteers) and 8-channel head coil (patients) and high-order shim. Functional images were acquired using a T2^*^-weighted BOLD sequence (repetition time/echo time = 2000/30 msec, matrix size = 64 × 64, field-of-view = 24 × 24 cm, slice thickness = 4 mm with no intersection gap, voxel size = 3.75 × 3.75 × 4 mm). This imaging protocol allowed adequate coverage of the entire brain. A high-resolution 3D spoiled gradient echo T1-weighted sequence was acquired for anatomic reference (repetition time/echo time = 6 msec/2 msec, matrix size = 256 × 256, field-of-view = 24 × 24 cm, slice thickness = 1.2 mm, with no intersection gap, voxel size = 0. 94 × 0.94 × 1.2 mm).

### Functional MRI paradigms

Three covert language paradigms were used: SSC, FAS, and CAT. All paradigms were block designed, with alternating active and control blocks. Each paradigm had a duration of 4 min and 20 seconds, with blocks alternating for 20 seconds each. All healthy participants and patients were exposed to the language paradigms in the same order. Prior to scanning, both healthy participants and cancer patients underwent practice trials as per our standard presurgical mapping guidelines to ensure that they can perform the task and generate adequate number of words. During functional imaging acquisition, the paradigm was displayed using an MR-compatible 32′ wide liquid crystal display, and oral instructions were provided via intercom.

#### Paradigm design

*Silent Sentence Completion*: Active block: The active block consisted of incomplete sentences for which participants had to fill in a blank. Each sentence was presented for 5 seconds, and each active block consisted of 4 different sentences.

Control block: Consisted of gibberish sentences with a format resembling the active block. The control block also consisted of 4 sentences, each lasting for 5 seconds.

*Word Generation and Category Naming*: FAS active block: Participants were presented with a letter of the alphabet and asked to generate words that started with the letter of alphabet shown. Each letter was presented for the entire 20 seconds.

CAT active block: Participants were presented with a category (e.g., cities, types of food, colors etc.) and asked to generate words related to the category shown. Each category was presented for the entire 20 seconds.

Control phase: In both FAS and CAT, participants were shown an image of an open hand and were asked to flex and extend their hands bilaterally, which was then simplified to opposing the first and the second digits bilaterally **(See attached supplementary material for complete paradigms).**

### Image Analysis

**Figure 1:**
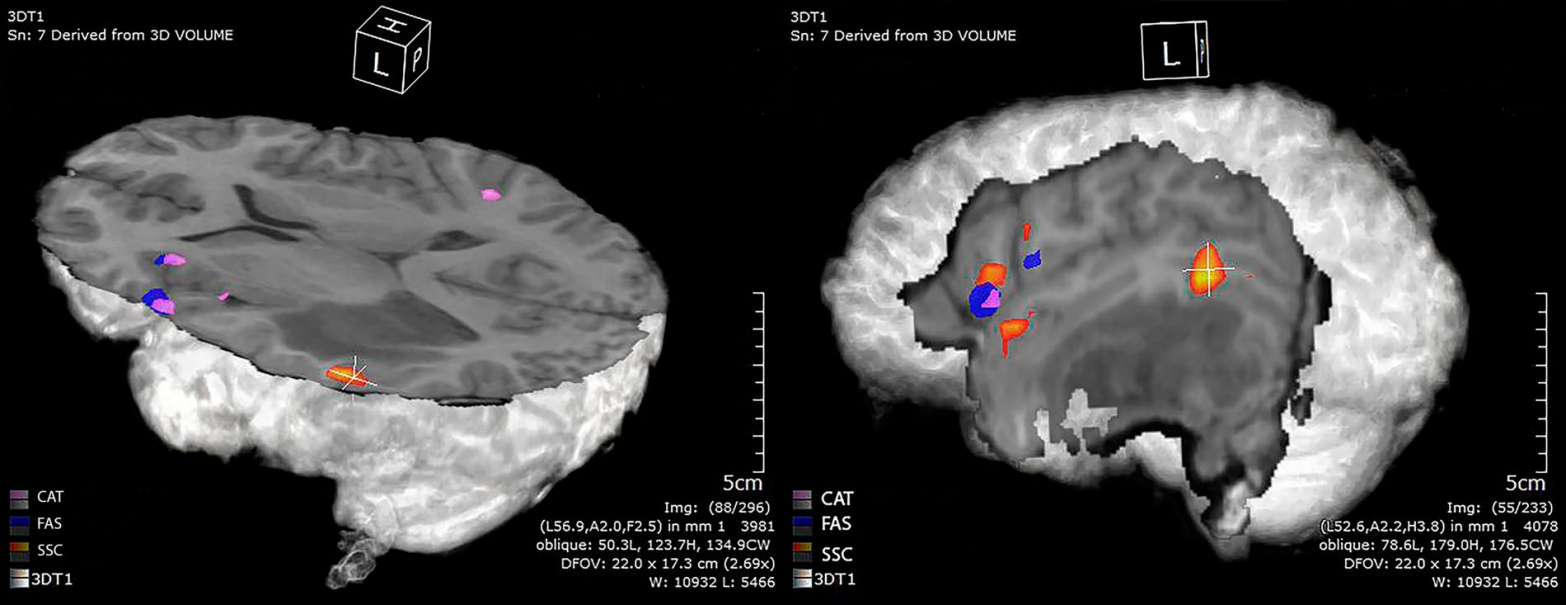
3D reconstruction of fMRI activation maps in a patient with left temporal lobe astrocytoma on SSC (Orange), FAS (Blue), and CAT (Violet).

All image analyses were performed using the Dynasuite Neuro 3.0 workstation (*In Vivo* Corporation, Gainesville, FL, USA). Image preprocessing included motion correction and spatial smoothing with a 4-mm full width at half maximum Gaussian kernel; no slice timing correction, signal was not de-trended. All of our analysis steps mimicked the clinical settings. The functional activation map was calculated using the correlation analysis between the task paradigm and the signal intensity time course of each voxel. A statistical threshold was applied to optimize visualization of language area (Fig. 1). Once all of these steps were performed, an ROI analysis was performed using the Dynasuite Neuro 3.0 workstation. First step included a manual thresholding of activation maps with a corrected pbonf-value of P ≤ 0.05. P-values were adjusted for each case and for each task separately in order to maximize the signal to noise ratio thus decrease the possibility of type II errors (false positive), which is crucial in the setting of preoperative mapping. Localization of activity in the Wernicke’s area was performed in-consensus by two experienced neuroradiologists (R.C.C., with 7 years of clinical experience with fMRI; A.J.K, with 15 years of experience with fMRI) blinded to the specific language paradigm. Activation in Wernicke’s area was defined as activation located in the temporal lobe, more specifically focused on the areas of the posterior superior temporal, middle temporal, and angular gyri, which correlate with the conventional anatomical location of centers of receptive speech processing. Activation was subsequently assessed on all 3 tasks for the presence or absence of activity. Activity in that specific area was assessed by using the fMRI probe tool on Dynasuite, which automatically incorporates that specific blob of activation into one homogenous ROI.

### Statistical analysis

#### Language Task Activation Rates

Activation rate is defined as the percentage/proportion of either cancer patients or healthy volunteers in which activation of Wernicke’s area was observed using a specific task (i.e. SSC, FAS, and CAT.) The activation rates of Wernicke’s area with SSC, FAS, and CAT were summarized using frequencies and percentages. The activation rate of SSC was reported with the mean, standard deviation, and range. McNemar’s test was used to compare the activation rate between the tasks.

#### Task Activation Profile and Tumor Genomics

Genomic analysis was performed after biopsy and the status of the following gene aberrations was collected: *TP53* (exon 8 mutation and immunohistochemistry [IHC]+), *IDH1* (exon 4 mutation and IHC+ for arginine [R] to a histidine [H] substitution at position 132 [p. R132H]), *IDH2* (exon 4 mutation or IHC+), *PTEN* (IHC+ or loss of wild-type expression), EGFR (IHC+), S100 (IHC+), *1p/19q* co-deletion, and *MGMT* promoter methylation^11,15,16^. Fisher’s exact test was used to assess the association between detection status (defined as presence or absence of functional activity) and genomic marker status for FAS and CAT. Wilcoxon rank sum test was used to assess the association between Extent of Activation (EOA) (number of voxels activated/total number of voxels in the region of interest [ROI]) and genomic marker status for SSC. All tests were 2-sided, and p-values of ≤0.05 were considered statistically significant. The statistical analysis was carried out using SAS software version 9.4 (SAS Institute, Cary, NC).

1 $$Extent\,of\,Activation=\frac{{\rm{Number}}\,{\rm{of}}\,{\rm{voxels}}\,{\rm{active}}}{{\rm{Total}}\,{\rm{number}}\,{\rm{of}}\,{\rm{voxels}}\,{\rm{in}}\,{\rm{ROI}}}$$

### Data availability

Most data generated or analysed during this study are included in the supplementary material. Additional data can be proided by the corresponding author upon reasonable request.

## Results

### Silent Sentence Completion vs Word Generation and Category Naming

#### Healthy Participants

SSC showed 100% activation of Wernicke’s area in healthy volunteers (Table 1), with bilateral activation in 1 volunteer. FAS and CAT detected significantly fewer cases in the healthy volunteer group than did SSC (p = 0.004 and 0.003 by McNemar’s test, respectively). For the same expected Wernicke’s region/ROI, FAS had a positive activation rate of 31% (5 of 16 cases), whereas CAT had a positive activation rate of 19% (3 of 16 cases). No significant difference was detected between FAS and CAT (p-value = 0.62 by McNemar’s test).

#### Patient Population

**Figure 2:**
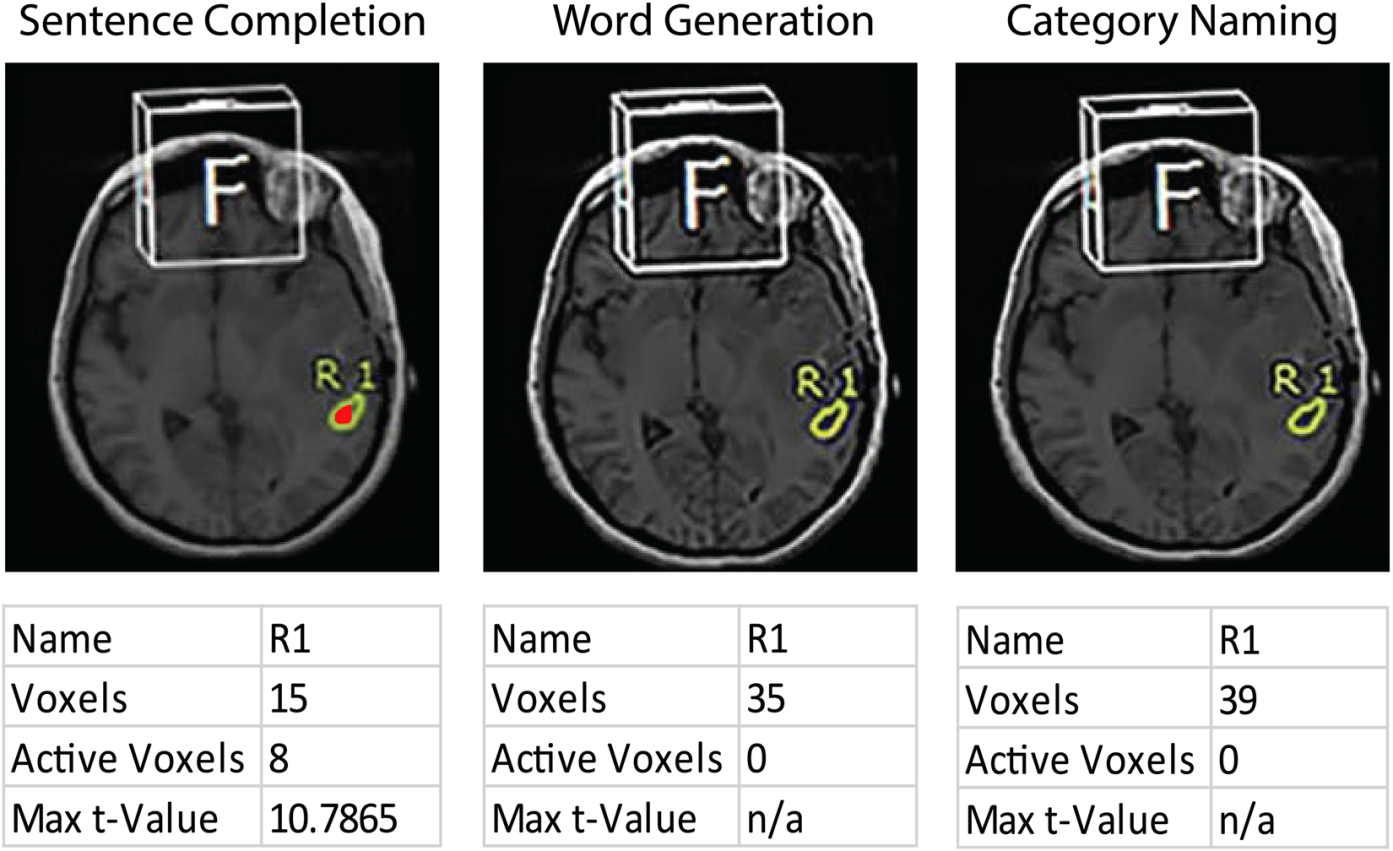
ROI (yellow outline) at the location of Wernicke’s area showing activity on SSC, FAS, and CAT, from left to right, respectively in a patient with left temporal glioblastoma. SSC showed 8 active voxels (red); no active voxels detected on FAS and CAT.

SSC had a 100% activation of Wernicke’s area in cancer patients (N = 35) (Table 2). For the same expected Wernicke’s region, FAS and CAT detected significantly less cases in the cancer group than did SSC (p FAS and CAT detected significantly fewer cases in the healthy volunteer group than did SSC (p < 0.0001 by McNemar’s test). FAS had a positive activation rate of 28.6% (10 of 35 cases), and CAT had a rate of 22.9% (8 of 35 cases) (Fig. 2). No significant difference was detected between FAS and CAT (p-value = 0.08 by McNemar’s test).

### Genomic markers and functional activation

There were statistically significant associations found between rates of functional activation and EOA in an ROI with genomic marker status in our cancer patients (Supplementary Tables 1–4). With regards to rates of functional activation, we found a significant association between *IDH1* mutation status and presence of functional activity on CAT. Out of our 35 patients, 15 (43%) patients had *IDH1* mutation. Patients with *IDH1* wild-type were more likely to be classified as negative on CAT; a higher percentage of patients with *IDH1* wild-type tumors showed no activation (90.48% versus 9.52%) versus those with *IDH1* mutation (60% versus 40%) (p-value = 0.05) (Table 4). However, this association did not replicate in the glioma histopathological subgroup analysis due to reduced sample size and the resulting smaller testing power. Functional activation was more common in patients with *IDH1* mutated versus *IDH1* wild-type tumors (40% versus 9.52%) (p-value = 0.05) (Table 4). This indicates that patients with *IDH1* mutated tumors have higher activation rates in Wernicke’s area. The latter trend was also seen on FAS. On the other hand, SSC showed constant activation in the Wernicke’s area regardless of *IDH1* mutation status or other genomic markers (Supplementary Table 5).

**Table 4:** Genomic markers and functional activation.

IDH-1 Mutation status and Wernicke’s activation on CAT					
	Activation on CAT				P-value (CAT)
	Negative		Positive		
IDH-1 Mutation	N	%	N	%	
Absent	19	90.48	2	9.52	0.05
Present	9	60.00	6	40.00	

Relating to EOA, there was a statistically significant association between SSC and EOA in patients with *1p/19q* co-deletion. In our cohort, 74% (26/35) of patients had *1p/19q* co-deletion. Patients without *1p/19q* co-deletion showed a higher median EOA compared with *1p/19q* co-deletion (71% versus 42%; p-value = 0.02) (Table 5). This finding indicates that patients with *1p/19q* co-deletion have a lower fraction of voxels activated within an ROI (milder level of activation) of Wernicke’s area. This association was not replicated after stratifying patients into their respective histopathological subgroups. No other significant associations were found between the other genomic markers and functional activation rate or EOA (Supplementary Tables 5 and 6).

**Table 5:** Genomic markers and functional activation.

ROI signal intensity on SSC and 1p/19q mutation status							
1p/19q co-deletion status	N	Mean	SD	Min	Median	Max	P-value
Absent	27	67%	22%	33%	71%	100%	0.02
Present	9	47%	13%	33%	42%	75%	

## Discussion

Our results clearly demonstrate that SSC is superior to FAS and CAT at localizing Wernicke’s area, with a significantly higher activation rate in both cancer patients and controls. Further, SSC showed those activation rates in Wernicke’s area across various types of gliomas in different anatomical locations, reflecting its efficacy and consistency. Our results also demonstrate that the presence of functional activation on SCC was constant and not affected by genomic mutation status. However, patients with *1p/19q* co-deletion showed less EOA in Wernicke’s area on SSC, and patients with *IDH1* mutation showed higher magnitude of signal intensity on CAT.

Given that our results revealed SSC to be a far more effective task in engaging Wernicke’s area and in the absence of any other receptive language paradigm that has been clinically tested on both healthy and brain tumor patients, we are confident that SSC can potentially be the task of choice when mapping receptive speech areas. As a reading comprehension task, SSC can activate areas in the posterior temporal cortex that pertain to language processing more comprehensively than all other verbal fluency or auditory word discrimination tasks^3^. Sentence completion requires simultaneous reading comprehension, semantic processing, and word retrieval, all of which demand neuronal processing in the superior, middle, and inferior temporal lobes^3,17,18^. Compared to the motor control phase of FAS and CAT, SSC’s control phase allowed the subtraction of any visual or semantic processing, thus increase the signal-to-noise ratio. As a covert task, SSC minimizes motion artifacts^19^, which usually contributes to increased signal noise, even after the deployment of image registration algorithms^20^. SSC is an adequate localizing and lateralizing task. In our study, lateralization was assessed qualitatively by the presence or absence of activation in the contralateral hemisphere, reproducing the clinical setting in which radiologists rely on their clinical judgment and expertise to determine lateralization. Including right-handed, left-handed, and ambidextrous patients ensured enough variation to confidently conclude that SSC is a robust lateralizing task. Bilateral activation, which was seen in one of the healthy participants, could be explained by the role that the right homologue of Wernicke’s area plays in the processing of subordinate meanings of words in healthy young individuals^21^.

As previously discussed, we found statistically significant associations between certain key genomic markers and functional activation. Patients with *IDH1* mutation had higher activation rates of Wernicke’s area on CAT. Patients with *1p*/*19q* non-codeletion demonstrated a higher magnitude of median and mean EOA on SSC. It is known that certain genetic aberrations affect the tumor microenvironment such as cerebral blood flow and peri-tumoral angiogenesis^11^, which can alter patterns of functional activity. In previous studies done on murine models, *IDH1* mutant mice exhibited an altered biochemical profile, which lead to a downstream increase in hypoxia inducible factor-1 α and its target genes including vascular endothelial growth factor^22,23^. This pattern of increased angiogenesis was also seen in a study by Kickingereder *et al*. (2015), who demonstrated that an increase in rCBV values increased the likelihood of an *IDH* mutant tumor^24^. In turn, this altered state of angiogenesis and cerebral perfusion could affect the BOLD signal and eventually translate to higher activation rates, which is concordant with our finding. Clinically, this also suggests that the tumoral genomic profile, if known, might eventually dictate the use of a specific batch of language tasks that have been shown to be more effective in activating specific cortical areas. On the other hand, even though there has been no direct correlation between 1p*/19q* co-deletion and tumoral angiogenesis, it has been associated with a higher relative CBV values^25^. This might be due decreased activation volumes because of NVU; a phenomenon that is dependent on tumor grade and type and on the distance to the eloquent cortex^26^. These findings highlight the potential of implementing genomic agnostic language paradigms and tailoring examinations according to a patient’s genomic profile, particularly in an era where the effectiveness of surgical and chemotheraputic interventions in the treatment of gliomas are influenced by a tumor’s genomic profile^27,28^.

This study included a relatively large number of healthy individuals and cancer patients, which provided two advantages: an adequate control group and robust statistical power. In addition, including healthy participants and cancer patients with various types of gliomas ensured the heterogeneity of our study population to account for most of the possible confounders that accompany an fMRI study and more importantly, account for the varying degrees of NVU across different types and grades of glioma^10^. The absence of correlation with direct brain cortical stimulation, which remains the gold standard for brain mapping^29^, is the main limitation of this study. We are currently in the process of conducting a study that aims to validate SSC via DCS and radiomic texture analysis. Another limitation stems from the controversy surrounding the classic anatomical model of language representation that describes Wernicke’s area as a discrete anatomical location, as opposed to the neurocognitive models, which describe receptive language centers as a complex interconnected network that is not limited to any specific location^3,30^. However, the aim of this study was primarily to show that SSC is, in fact, robust for the identification of important cortical areas of receptive language processing, which is necessary for function-preserving surgery, rather than to delineate the relationship between different areas of language processing^31^. Furthermore, in this study we compared SSC to CAT and FAS, which are the tasks most frequently used in the clinic for language mapping; other tasks that require language comprehension have been recently proposed, thus further research comparing SSC with these tasks is warranted.

In conclusion, SCC is a highly effective paradigm for activating Wernicke’s area. The paradigm is superior to the widely used FAS and CAT paradigms, providing a reliable clinical tool for preoperative brain mapping. In addition, there seems to be an association between tumor genomics and patterns of functional activity. This might translate as variations in task-related activation rates and EOA seen in eloquent cortex, an aspect that is extremely valuable in the setting of preoperative mapping and should be investigated further.

## Electronic supplementary material

Supplementary Material
