# Supplementary material for: Silent Sentence Completion Shows Superiority Localizing Wernicke’s Area and Activation Patterns of Distinct Language Paradigms Correlate with Genomics: Prospective Study
Source: Sci Rep. 2017 Sep 21;7:12054. doi: 10.1038/s41598-017-11192-2 (PMC5608896; doi:10.1038/s41598-017-11192-2)
Supplement: Supplementary file 1 — Supplementary Material [file 41598_2017_11192_MOESM1_ESM.doc]

**Silent Sentence Completion Shows Superiority Localizing Wernicke’s Area and Activation Patterns of Distinct Language Paradigms Correlate with Genomics: Prospective Study**

Kamel El Salek^1^; Islam S. Hassan^1^; Aikaterini Kotrotsou^1,9^; Srishti Abrol^1^; Scott H. Faro^2^; Feroze B. Mohamed^3^; Pascal O. Zinn^4^; Wei Wei^5^; Nan Li^5^; Ashok J. Kumar^1^; Jeffrey S. Weinberg^6^; Jeffery S. Wefel^7^; Shelli R. Kesler^7^; Ho-Ling Anthony Liu^8^; Ping Hou^8^; R. Jason Stafford^8^; Sujit Prabhu^6^; Raymond Sawaya^6^; Rivka R. Colen^1,9*^

^1^Section of Neuroradiology, Department of Diagnostic Radiology, The University of Texas MD Anderson Cancer Center, Houston, Texas.

^2^Department of Radiology, Temple University, Philadelphia, Pennsylvania.

^3^Thomas Jefferson University Hospital, Philadelphia, Pennsylvania.

^4^Department of Neurosurgery, Baylor College of Medicine, Houston, Texas.

^5^Division of Quantitative Sciences, Department of Biostatistics, The University of Texas MD Anderson Cancer Center, Houston, Texas.

^6^Department of Neurosurgery, The University of Texas MD Anderson Cancer Center, Houston, Texas.

^7^Section of Neuropsychology, Department of Neuro-Oncology, Division of Cancer Medicine, The University of Texas MD Anderson Cancer Center, Houston, Texas.

^8^Department of Imaging Physics, The University of Texas MD Anderson Cancer Center, Houston, Texas. ^9^Department of Cancer Systems Imaging, The University of Texas MD Anderson Cancer Center, Houston, Texas.,

**Corresponding Author:**

Rivka R. Colen, M.D.
Assistant Professor

Department of Cancer Systems Imaging and Diagnostic Radiology

The University of Texas MD Anderson Cancer Center

1881 East Road, 3SCRB4.3606

Houston, TX 77054-1907

Telephone: (713)-745-8552

Fax: (713)-794-5456

E-mail: rcolen@mdanderson.org

**Supplementary Material**

| **Patient** | **SSC** | **FAS** | **CAT** | ***IDH-1*** | ***IDH-2*** | ***PTEN*** | **EGFR** | ***TP53*** | ***s-100*** | ***1p/19q***  ***co-del*** | ***MGMT* Promoter methylation** | **Pathology** | **% act SSC** | **% act FAS** | **% act CAT** |
| --- | --- | --- | --- | --- | --- | --- | --- | --- | --- | --- | --- | --- | --- | --- | --- |
| 1 | Pos | Neg | Neg | A | A | P | P | P | A | A | A | GBM | 50 | 0 | 0 |
| 2 | Pos | Neg | Neg | P | A | A | P | A | A | P | NT | Oligodendroglioma | 50 | 0 | 0 |
| 3 | Pos | Pos | Pos | A | A | A | P | A | A | A | A | GBM | 79 | 5.5 | 14 |
| 4 | Pos | Pos | Neg | A | A | A | P | A | A | A | A | GBM | 33 | 0 | 0 |
| 5 | Pos | Pos | Pos | P | A | A | A | A | A | P | A | Oligodendroglioma | 33 | 11 | 60 |
| 6 | Pos | Neg | Neg | A | A | A | P | P | A | A | NT | GBM | 50 | 0 | 0 |
| 7 | Pos | Neg | Neg | P | A | A | P | P | A | A | NT | Mixed oligoastrocytoma | 71 | 0 | 0 |
| 8 (ROI-1) | Pos | Neg | Neg | A | A | A | P | A | A | A | P | GBM | 37.5 | 0 | 0 |
| 8 (ROI-2) | Pos | Neg | Neg | A | A | A | P | A | A | A | P | GBM | 100 | 0 | 0 |
| 9 | Pos | Neg | Neg | A | A | A | A | P | A | A | NT | GBM | 88 | 0 | 0 |
| 10 | Pos | Neg | Neg | P | A | A | A | A | A | P | NT | Oligodendroglioma | 75 | 0 | 0 |
| 11 | Pos | Neg | Neg | A | A | P | P | A | A | A | NT | GBM | 75 | 0 | 0 |
| 12 | Pos | Neg | Neg | A | A | A | A | A | A | A | NT | GBM | 87.5 | 0 | 0 |
| 13 | Pos | Neg | Neg | P | A | A | A | P | A | A | NT | GBM | 100 | 0 | 0 |
| 14 | Pos | Neg | Neg | P | A | A | A | A | A | P | NT | Oligodendroglioma | 44 | 0 | 0 |
| 15 | Pos | Neg | Neg | P | A | A | A | A | A | P | A | Oligodendroglioma | 37.5 | 0 | 0 |
| 16 | Pos | Neg | Neg | P | A | A | A | A | A | P | NT | Oligodendroglioma | 40 | 0 | 0 |
| 17 | Pos | Neg | Neg | A | A | A | A | P | A | A | NT | GBM | 71 | 0 | 0 |
| 18 | Pos | Neg | Neg | A | A | A | A | p | A | A | NT | GBM | 50 | 0 | 0 |
| 19 | Pos | Pos | Pos | P | A | A | A | P | A | A | NT | Astrocytoma | 37.5 | 45 | 89 |
| 20 | Pos | Neg | Neg | A | A | A | A | P | p | A | NT | GBM | 100 | 0 | 0 |
| 21 | Pos | Neg | Neg | A | A | A | A | A | P | A | NT | GBM | 53 | 0 | 0 |
| 22 | Pos | Neg | Neg | A | A | A | A | P | A | A | NT | GBM | 100 | 0 | 0 |
| 23 | Pos | Pos | Pos | P | A | A | P | A | A | A | NT | Astrocytoma | 84 | 38.4 | 20 |
| 24 | Pos | Pos | Pos | A | A | P | P | P | A | A | NT | GBM | 83 | 25 | 71 |
| 25 | Pos | Neg | Pos | A | A | A | A | A | A | A | NT | Ganglioglioma | 67 | 0 | 0 |
| 26 | Pos | Neg | Neg | A | A | P | P | P | A | A | NT | GBM | 57 | 0 | 0 |
| 27 | Pos | Neg | Neg | A | A | A | A | P | A | A | A | GBM | 57 | 0 | 0 |
| 28 | Pos | Neg | Neg | P | A | A | A | A | A | A | NT | Astrocytoma | 37.5 | 0 | 0 |
| 29 | Pos | Pos | Pos | P | A | A | A | A | A | P | NT | Oligodendroglioma | 41.6 | 33 | 41.6 |
| 30 | Pos | Pos | Neg | A | A | A | A | A | A | A | NT | Oligodendroglioma | 40 | 26 | 0 |
| 31 | Pos | Pos | Neg | A | P | A | A | A | A | P | NT | Oligodendroglioma | 60 | 40 | 0 |
| 32 | Pos | Neg | Neg | A | A | A | A | P | A | A | NT | Astrocytoma | 50 | 0 | 0 |
| 33 | Pos | Pos | Pos | P | A | A | A | A | A | A | NT | Astrocytoma | 75 | 23 | 7 |
| 34 | Pos | Pos | Pos | P | A | A | A | A | A | P | NT | Diffuse glioma | 40 | 30 | 42 |
| 35 | Pos | Neg | Neg | P | A | A | A | A | A | A | NT | Astrocytoma | 83 | 0 | 0 |

***Table 1: Overall distribution of genetic aberrations, tumor type, and percentage activation***

***A=absent, P=present, NT=not tested.***

| **SSC** | **FAS** | **CAT** | ***IDH-1*** | ***IDH-2*** | ***PTEN*** | **EGFR** | ***TP53*** | ***s-100*** | ***1p/19q co-del*** | ***MGMT* promoter methylation** | **% act SSC** | **% act FAS** | **% act CAT** |
| --- | --- | --- | --- | --- | --- | --- | --- | --- | --- | --- | --- | --- | --- |
| Pos | Neg | Neg | A | A | P | P | A | A | A | A | 50 | 0 | 0 |
| Pos | Pos | Pos | A | A | A | P | A | A | A | A | 79 | 5.5 | 14 |
| Pos | Pos | Neg | A | A | A | P | A | A | A | A | 33 | 0 | 0 |
| Pos | Neg | Neg | A | A | A | P | P | A | A | NT | 50 | 0 | 0 |
| Pos | Neg | Neg | A | A | A | P | A | A | A | P | 37.5 | 0 | 0 |
| Pos | Neg | Neg | A | A | A | P | A | A | A | P | 100 | 0 | 0 |
| Pos | Neg | Neg | A | A | A | A | P | A | A | NT | 88 | 0 | 0 |
| Pos | Neg | Neg | A | A | P | P | A | A | A | NT | 75 | 0 | 0 |
| Pos | Neg | Neg | A | A | A | A | A | A | A | NT | 87.5 | 0 | 0 |
| Pos | Neg | Neg | P | A | A | A | P | A | A | NT | 100 | 0 | 0 |
| Pos | Neg | Neg | A | A | A | A | P | A | A | NT | 71 | 0 | 0 |
| Pos | Neg | Neg | A | A | A | A | P | A | A | NT | 50 | 0 | 0 |
| Pos | Neg | Neg | A | A | A | A | P | P | A | NT | 100 | 0 | 0 |
| Pos | Neg | Neg | A | A | A | A | A | P | A | NT | 53 | 0 | 0 |
| Pos | Neg | Neg | A | A | A | A | P | A | A | NT | 100 | 0 | 0 |
| Pos | Pos | Pos | A | A | P | P | P | A | A | NT | 83 | 25 | 71 |
| Pos | Neg | Neg | A | A | P | P | P | A | A | NT | 57 | 0 | 0 |
| Pos | Neg | Neg | A | A | A | A | P | A | A | A | 57 | 0 | 0 |

***Table 2: Genetic aberrations and activation rates among GBM patients***

***A=absent, P=present, NT=not tested.***

***Table 3: Genetic aberrations and activation rates among oligodendroma patients***

| **SSC** | **FAS** | **CAT** | ***IDH-1*** | ***IDH-2*** | ***PTEN*** | **EGFR** | ***TP53*** | ***S-100*** | **1p/19q co-del** | ***MGMT* promoter methylation** | **% act SSC** | **% act FAS** | **% act CAT** |
| --- | --- | --- | --- | --- | --- | --- | --- | --- | --- | --- | --- | --- | --- |
| Positive | Negative | Negative | P | A | A | P | A | A | P | NT | 50 | 0 | 0 |
| Positive | Positive | Positive | P | A | A | A | A | A | P | A | 30 | 11 | 60 |
| Positive | Negative | Negative | P | A | A | A | A | A | P | NT | 75 | 0 | 0 |
| Positive | Negative | Negative | P | A | A | A | A | A | P | NT | 44 | 0 | 0 |
| Positive | Negative | Negative | P | A | A | A | A | A | P | A | 37.5 | 0 | 0 |
| Positive | Negative | Negative | P | A | A | A | A | A | P | NT | 40 | 0 | 0 |
| Positive | Positive | Positive | P | A | A | A | A | A | P | NT | 41.6 | 33 | 41.6 |
| Positive | Positive | Negative | A | A | A | A | A | A | A | NT | 40 | 26 | 0 |
| Positive | Positive | Negative | A | P | A | A | A | A | P | NT | 60 | 40 | 0 |

***A=absent, P=present, NT=not tested.***

***Table 4:*** ***Genetic aberrations and activation rates among astrocytoma patients***

| **SSC** | **FAS** | **CAT** | ***IDH-1*** | ***IDH-2*** | ***PTEN*** | **EGFR** | ***TP53*** | ***S-100*** | ***1p/19q co-del*** | ***MGMT* promoter methylation** | **% act SSC** | **% act FAS** | **% act CAT** |
| --- | --- | --- | --- | --- | --- | --- | --- | --- | --- | --- | --- | --- | --- |
| Positive | Positive | Positive | P | A | A | A | P | A | A | NT | 37.5 | 45 | 89 |
| Positive | Positive | Positive | P | A | A | P | A | A | A | NT | 84 | 38.4 | 20 |
| Positive | Negative | Negative | P | A | A | A | A | A | A | NT | 37.5 | 0 | 0 |
| Positive | Negative | Negative | A | A | A | A | P | A | A | NT | 50 | 0 | 0 |
| Positive | Positive | Positive | P | A | A | A | A | A | A | NT | 75 | 23 | 7 |
| Positive | Negative | Negative | P | A | A | A | A | A | A | NT | 83 | 0 | 0 |

***A=absent, P=present, NT=not tested.***

| **Table 5. Summary of mutation status by imaging finding (Detection/Activation rate)** | | | | | | | | | | | | |
| --- | --- | --- | --- | --- | --- | --- | --- | --- | --- | --- | --- | --- |
|  | **SSC** | | **FAS** | | | | **p-value (FAS)** | **CAT** | | | | **P-value (CAT)** |
|  | **pos** | | **neg** | | **pos** | |  | **neg** | | **pos** | |  |
|  | **N** | **%** | **N** | **%** | **N** | **%** |  | **N** | **%** | **N** | **%** |  |
| **IDH1** |  | | | | | | | | | | | |
| **0** | 21 | 100.00 | 16 | 76.19 | 5 | 23.81 | 0.46 | 19 | 90.48 | 2 | 9.52 | 0.05 |
| **1** | 15 | 100.00 | 9 | 60.00 | 6 | 40.00 |  | 9 | 60.00 | 6 | 40.00 |  |
| **PTEN** |  | | | | | | | | | | | |
| **0** | 32 | 100.00 | 22 | 68.75 | 10 | 31.25 | 1 | 25 | 78.13 | 7 | 21.88 | 1 |
| **1** | 4 | 100.00 | 3 | 75.00 | 1 | 25.00 |  | 3 | 75.00 | 1 | 25.00 |  |
| **EGFR** |  | | | | | | | | | | | |
| **0** | 24 | 100.00 | 17 | 70.83 | 7 | 29.17 | 1 | 19 | 79.17 | 5 | 20.83 | 1 |
| **1** | 12 | 100.00 | 8 | 66.67 | 4 | 33.33 |  | 9 | 75.00 | 3 | 25.00 |  |
| **P53** |  | | | | | | | | | | | |
| **0** | 22 | 100.00 | 13 | 59.09 | 9 | 40.91 | 0.14 | 16 | 72.73 | 6 | 27.27 | 0.44 |
| **1** | 14 | 100.00 | 12 | 85.71 | 2 | 14.29 |  | 12 | 85.71 | 2 | 14.29 |  |
| **s100** |  | | | | | | | | | | | |
| **0** | 34 | 100.00 | 23 | 67.65 | 11 | 32.35 | 1 | 26 | 76.47 | 8 | 23.53 | 1 |
| **1** | 2 | 100.00 | 2 | 100.00 | 0 | 0 |  | 2 | 100.00 | 0 | 0 |  |
| **1p19q** |  | | | | | | | | | | | |
| **0** | 27 | 100.00 | 20 | 74.07 | 7 | 25.93 | 0.41 | 22 | 81.48 | 5 | 18.52 | 0.38 |
| **1** | 9 | 100.00 | 5 | 55.56 | 4 | 44.44 |  | 6 | 66.67 | 3 | 33.33 |  |
| **IDH2** |  | | | | | | | | | | | |
| **0** | 35 | 100.00 | 25 | 71.43 | 10 | 28.57 | 0.31 | 27 | 77.14 | 8 | 22.86 | 1 |
| **1** | 1 | 100.00 | 0 | 0 | 1 | 100.00 |  | 1 | 100.00 | 0 | 0 |  |
| **MGMT** |  | | | | | | | | | | | |
| **0** | 6 | 100.00 | 3 | 40.00 | 3 | 60.00 | 0.46 | 4 | 66.67 | 2 | 33.33 | >0.99 |
| **1** | 2 | 100.00 | 2 | 100.00 | 0 | 0 |  | 2 | 100.00 | 0 | 0 |  |

| **Table 6. Association of mutation status with Signal Intensity on SSC in all patients.** | | | | | | | |
| --- | --- | --- | --- | --- | --- | --- | --- |
|  | **N** | **Mean** | **SD** | **Min** | **Median** | **Max** | **p-value** |
| **IDH1** |  | | | | | |  |
| **0** | 21 | 0.66 | 0.21 | 0.33 | 0.60 | 1.00 | 0.14 |
| **1** | 15 | 0.57 | 0.22 | 0.33 | 0.44 | 1.00 |  |
| **PTEN** |  | | | | | | |
| **0** | 32 | 0.62 | 0.23 | 0.33 | 0.55 | 1.00 | 0.54 |
| **1** | 4 | 0.66 | 0.15 | 0.50 | 0.66 | 0.83 |  |
| **EGFR** |  | | | | | | |
| **0** | 24 | 0.61 | 0.23 | 0.33 | 0.55 | 1.00 | 0.69 |
| **1** | 12 | 0.64 | 0.21 | 0.33 | 0.64 | 1.00 |  |
| **P53** |  | | | | | | |
| **0** | 22 | 0.58 | 0.21 | 0.33 | 0.52 | 1.00 | 0.12 |
| **1** | 14 | 0.69 | 0.22 | 0.38 | 0.64 | 1.00 |  |
| **s100** |  | | | | | | |
| **0** | 34 | 0.61 | 0.21 | 0.33 | 0.57 | 1.00 | 0.32 |
| **1** | 2 | 0.77 | 0.33 | 0.53 | 0.77 | 1.00 |  |
| **1p/19q** |  | | | | | |  |
| **0** | 27 | 0.67 | 0.22 | 0.33 | 0.71 | 1.00 | 0.02 |
| **1** | 9 | 0.47 | 0.13 | 0.33 | 0.42 | 0.75 |  |
| **IDH2** |  | | | | | | |
| **0** | 35 | 0.62 | 0.22 | 0.33 | 0.57 | 1.00 | 0.88 |
| **1** | 1 | 0.60 | 0 | 0.60 | 0.60 | 0.60 |  |
| **MGMT** |  | | | | | | |
| **0** | 6 | 48.25 | 17.92 | 33.00 | 43.75 | 79.00 | 0.50 |
| **1** | 2 | 68.75 | 44.19 | 37.50 | 68.75 | 100.00 |  |

**Tasks:**

1. Sentence Completion Task
2. Crying babies make a lot of _____.
3. Lions have very sharp ____.
4. Soup should be eaten with a _____.
5. Whales and dolphins live in the ____.
6. Crying babies make a lot of _____.
7. She likes to eat chocolate chip _____.
8. When the light turns green you ____.
9. A triangle has three _____.
10. Before going to bed she brushed her _____.
11. At the deep end the pool had a diving ____.
12. On the beach there is a lot of _____.
13. When it's hot out I like to eat ______.
14. There is no school on Saturday or _____.
15. He wrote a note on a piece of _____.
16. When the lights go out it is _____.
17. Humpty Dumpty sat on a _____.
18. The opposite of inside is _____.
19. I keep my money in a _____.
20. To make purple you mix red and ____.
21. If you eat too much you will be ______.
22. Astronauts use rockets to go to outer ______.
23. A baseball game has nine _____.
24. He ate a peanut butter and jelly _____.
25. Cats purr when they are ______.
26. If you leave ice cream in the sun it will ____.
27. My favorite movie is _______.
28. For Halloween he carved a big orange ______.
29. The dog barked when it was _____.
30. My mother's name is _____.
31. MacDonald's sells lots of French _____.
32. She went to Disney World and saw Mickey ____.
33. Letters:

S, T, L, C, M, A

1. Categories:
2. Animals
3. Food
4. Beverages
5. Animals
6. Beverages
7. Parts of the Body
8. Cities
